# Monounsaturated fatty acids, olive oil and health status: a systematic review and meta-analysis of cohort studies

**DOI:** 10.1186/1476-511X-13-154

**Published:** 2014-10-01

**Authors:** Lukas Schwingshackl, Georg Hoffmann

**Affiliations:** Department of Nutritional Sciences, Faculty of Life Sciences, University of Vienna, Althanstraße 14 (UZAII), A-1090 Vienna, Austria

**Keywords:** Monounsaturated fatty acids, Olive oil, Cohort studies, Meta-analysis, Cardiovascular disease

## Abstract

**Background:**

The aim of the present meta-analysis of cohort studies was to focus on monounsaturated fat (MUFA) and cardiovascular disease, cardiovascular mortality as well as all-cause mortality, and to distinguish between the different dietary sources of MUFA.

**Methods:**

Literature search was performed using the electronic databases PUBMED, and EMBASE until June 2nd, 2014. Study specific risk ratios and hazard ratios were pooled using a inverse variance random effect model.

**Results:**

Thirty-two cohort studies (42 reports) including 841,211 subjects met the objectives and were included. The comparison of the top versus bottom third of the distribution of a combination of MUFA (of both plant and animal origin), olive oil, oleic acid, and MUFA:SFA ratio in each study resulted in a significant risk reduction for: all-cause mortality (RR: 0.89, 95% CI 0.83, 0.96, p = 0.001; I^2^ = 64%), cardiovascular mortality (RR: 0.88, 95% CI 0.80, 0.96, p = 0.004; I^2^ = 50%), cardiovascular events (RR: 0.91, 95% CI 0.86, 0.96, p = 0.001; I^2^ = 58%), and stroke (RR: 0.83, 95% CI 0.71, 0.97, p = 0.02; I^2^ = 70%). Following subgroup analyses, significant associations could only be found between higher intakes of olive oil and reduced risk of all-cause mortality, cardiovascular events, and stroke, respectively. The MUFA subgroup analyses did not reveal any significant risk reduction.

**Conclusion:**

The results indicate an overall risk reduction of all-cause mortality (11%), cardiovascular mortality (12%), cardiovascular events (9%), and stroke (17%) when comparing the top versus bottom third of MUFA, olive oil, oleic acid, and MUFA:SFA ratio. MUFA of mixed animal and vegetable sources per se did not yield any significant effects on these outcome parameters. However, only olive oil seems to be associated with reduced risk. Further research is necessary to evaluate specific sources of MUFA (i.e. plant vs. animal) and cardiovascular risk.

**Electronic supplementary material:**

The online version of this article (doi:10.1186/1476-511X-13-154) contains supplementary material, which is available to authorized users.

## Background

The most common monounsaturated fatty acids (MUFA) in daily nutrition is oleic acid, followed by palmitoleic acid, and vaccenic acid. Moreover, oleic acid represents the topmost MUFA provided in the diet (~90% of all MUFA). No dietary recommendations for MUFA are given by the National Institute of Medicine, the United States Department of Agriculture, the European Food and Safety Authority and the American Diabetes Association. In contrast, the Academy of Nutrition and Dietetics as well as the Canadian Dietetic Association both promote <20% MUFA of daily total energy consumption, while the American Heart Association sets a limit of 20% MUFA in their respective guidelines [1–3]. One reason for specific MUFA recommendations might be their potential benefit in the primary and secondary prevention of cardiovascular diseases. However, previous meta-analyses of cohort studies reported inconsistent results of MUFA on coronary heart disease (CHD). Jakobsen et al. [4] observed that replacement of SFA by MUFA marginally increased the risk of coronary events, whereas no significant effects on coronary death could be observed. These results are in strong discrepancy with another meta-analysis of cohort studies, were Mente et al. [5] reported a significant correlation between MUFA intake and a decrease in the relative risk for CHD. Skeaff and Miller [6] did not observe any effects of MUFA-rich diets on relative risks of CHD events and CHD death. Likewise, the most recent meta-analysis by Chowdhury et al. including nine cohort studies found no significant associations between MUFA intake, circulating MUFA and risk of CHD [7].

One explanation for these inconclusive data might be that different sources of MUFA were not taken into account. Adopting a western diet means that MUFA is predominantly supplied by foods of animal origin, while in south European countries, extra virgin olive oil is the most dominant source of this type of fatty acid [8]. Results of the recently published PREDIMED trial demonstrated major cardiovascular benefits of olive oil and nuts when compared to a low-fat diet [9]. As a major outcome parameter, the risk of stroke was reduced, an event which has not been included in the meta-analyses mentioned above. In addition, a recent cohort study observed a significant association between dietary olive oil, higher plasma oleic acid and reduced risk of stroke [10]. Extra virgin olive oil is regarded to be the genuine driver of the Mediterranean diet and was found to be associated with a 26% reduced risk of all-cause mortality in the Spanish branch of the EPIC study [11]. The aim of the present meta-analysis of cohort studies was to focus on MUFA and CVD (combining CHD and stroke), cardiovascular mortality, and all-cause mortality, and to distinguish between the different dietary sources of MUFA (e.g. olive oil).

## Materials and methods

### Literature search

Queries of literature were performed using the electronic databases PUBMED, and EMBASE (until 2nd June 2014, respectively) with no restrictions to language, and calendar date using the following search terms: *(“dietary fat” OR “fatty acids” OR “monounsaturated fat” OR “mufa” OR “olive oil” OR “oleic acid” OR “mediterranean diet”) AND (“cardiovascular disease” OR “myocardial infarction” OR “coronary heart disease” OR “stroke” OR “mortality”) AND (“incidence” OR “cohort” OR “follow-up” OR “prospective” OR “risk ratio” OR “hazard ratio” OR “rate ratio”).* Moreover, the reference lists from retrieved articles, systematic reviews, and meta-analyses were checked to search for further relevant studies. This systematic review was planned, conducted, and reported in adherence to standards of quality for reporting meta-analyses [12]. Literature search was conducted independently by both authors, with disagreements resolved by consensus.

### Eligibility criteria

Studies were included in the meta-analysis if they met all of the following criteria: *(i)* cohort study design; *(ii)* data related to dietary consumption of MUFA, MUFA:SFA ratio, olive oil, and oleic acid; *(iii)* the primary outcomes were: all-cause mortality, CVD mortality, combined CVD events (cardiovascular mortality, cardiovascular morbidity (non-fatal myocardial infarction, angina, stroke, heart failure, peripheral vascular events)); the secondary outcomes were: coronary heart disease, and stroke; *(iv)* adjusted relative risks (RRs), and hazard ratios (HRs) with corresponding 95% confidence intervals (95% CIs) or the data necessary to calculate these; *(v)* when a study appeared to have been published in duplicate, the version containing the most comprehensive information was selected.

### Data extraction and quality assessment

The following data were extracted from each study: the first author’s last name, year of publication, study origin, outcome parameter, sample size, study length, age at entry, sex, specification of MUFA, adjustment factors, quality score, and risk estimates (HR, RR; highest vs. lowest category) with their corresponding 95% CIs. If separate risk estimates for males and females or separate risk estimates for ages were available in one study, the data were pooled and treated as one study. When a study provided several risk estimates, the maximally adjusted model was chosen. To assess the study quality, a 9-point scoring system according to the Newcastle-Ottawa Scale (NOS) was used. Hence, the full score was 9, and a high-quality study in the present analysis was defined by a threshold of ≥ 7 points [13]. Data extraction and quality assessment were performed by one author (L.S).

### Statistical analysis

The meta-analysis was performed by combining the multivariable adjusted RR or HR of the highest compared with the lowest MUFA, MUFA:SFA ratio, oleic acid, or olive oil category based on random effects model using DerSimonian-Laird method, which incorporated both within and between study variability [14]. To ensure a transparent approach to meta-analysis and interpretation of findings in this review, RR/HR estimates for association of fatty acids and primary/secondary outcomes that were often differently reported by each study (such as per-unit or per-1-SD change or comparing quintiles, quartiles, thirds, and other groupings) were transformed, using methods previously described [7]. These transformed estimates consistently corresponded to the comparison of the top versus bottom third of MUFA, MUFA:SFA ratio, olive oil, and oleic acid distribution in each study. To evaluate the weighting of each study, the standard error for the logarithm HR/RR of each study was calculated and regarded as the estimated variance of the logarithm HR/RR using an inverse variance method [14]. Studies were grouped according to the different clinical outcomes (all-cause mortality, cardiovascular mortality, combined cardiovascular events, coronary heart disease, and stroke). Subgroup analysis was performed for total MUFA, MUFA:SFA ratio, oleic acid, and olive oil. Heterogeneity was estimated by the Cochrane Q test together with the I^2^ statistic. An I^2^ value >50% indicates substantial heterogeneity across studies [15]. The *heterogi* command in STATA was used to calculate the confidence intervals for the heterogeneity estimates. Funnel plots were used to assess potential publication bias. To determine the presence of publication bias, we assessed the symmetry of the funnel plots in which mean differences were plotted against their corresponding standard errors. In addition, Egger test was performed to test for potential publication bias [16]. Sensitivity analyses were performed assuming statistical heterogeneity with the *metaan* command in STATA [17]. All analyses were conducted using the Review Manager by the Cochrane Collaboration (version 5.2) and STATA 13.0 (Stata-Corp, College Station, TX).

### Missing data

Dr. Goldbourt (personal communication) provided the 23 year follow-up all-cause mortality and cardiovascular mortality data of the Israeli civil cohort for the highest vs. lowest quintile MUFA: SFA ratio [18].

## Results

### Literature search and study characteristics

A total of 32 cohort studies (42 reports) met the inclusion criteria and were included in the meta-analysis [10, 11, 18–57]. Full search strategy for PUBMED is given in the Additional file 1. General study characteristics are given in Table 1. Sample size varied between 161 and 161,808 with a follow-up time ranging from 3.7 to 30 years. The total number of subjects in the included studies was 841,211.

**Table 1:** **General study characteristics of the included cohort studies**

Author, year	Cohort name country	Outcomes	Population	Follow-up (years)	Age at entry (years)	Sex	MUFA source	Adjustment	Multivariate adjusted	Study quality
										(Newcastle-Ottawa scale)
Atkins et al. 2014 [34]	British Regional Heart Study GBR	All-cause mortality CVD mortality CVD events CHD events	3,328	11.3	60-79	M	Olive oil	Age, energy intake, smoking, alcohol, PA, social class, BMI, and a modified version of the HDI/EDI score not containing the individual component of interest	Fourth vs. first quartile	8
Barzi et al. 2003 [50]	Studio della Sopravvivenza nell’Infarto Miocardico ITA	All-cause mortality	11,323 with myocardial infarction	6.5	59	M/F	Olive oil	Age, sex, hypertension, HDL-cholesterol, diabetes, smoking, claudication, electrical instability, left ventricular dysfunction, residual myocardial ischaemia, dietary supplementation, pharmacological therapies	Third vs. first tertile	7
Bendinelli et al. 2011 [49]	European Prospective Into Cancer and Nutrition ITA	CHD	29,689	7.85	35-74	F	MUFA MUFA:SFA	Energy intake, educational level, smoking status, alcohol consumption, body height, body weight, waist circumference daily non-alcohol caloric intake, hypertension, menopausal status, PA, total meat consumption	Fourth vs. first quartile	8
Buckland et al. 2012 [11]	European Prospective Into Cancer and Nutrition SPA	All-cause mortality CVD mortality Cancer mortality	41,078	10.4	29-69	M/F	Olive oil	Centre, sex, age, energy intake, BMI, waist circumference, educational status, smoking status, PA, and alcohol intake, intake of fruit, vegetables, meat, and dairy	Fourth vs. first quartile	8
Buckland et al. 2012 [56]	European Prospective Into Cancer and Nutrition SPA	CHD	40,142	10.4	29-69	M/F	Olive oil	Educational level, BMI, waist circumference, PA, smoking status, alcohol consumption, energy intake excluding alcohol, hyperlipidaemia, hypertension and diabetes, Mediterranean diet score (excluding olive oil and alcohol)	Fourth vs. first quartile	8
Chiuve et al. 2012 [32]	Nurses’ Health Study USA	Sudden death	91,981	30	34-59	F	MUFA	Total calories, smoking, BMI, family history of myocardial infarction, menopausal status, hormone therapy, exercise, aspirin use, use of multivitamins, use of vitamin E supplements, alcohol use, and history of diabetes, hypertension, hypercholesterolemia, coronary heart disease, and cancer at baseline, percentage of energy from total fat	Fifth vs. first quintile	8
Dilis et al. 2012 [19]	European Prospective Into Cancer and Nutrition GRE	CHD mortality CHD incidence	23,929	10	20-86	M/F	MUFA Olive Oil MUFA:SFA	Age, BMI, height, PA, years of schooling and energy intake entered, alcohol consumption, smoking status and arterial blood pressure	1 SD increment	9
Esrey et al. 1996 [42]	Lipid Research Clinics Prevalence Study USA	CVD mortality	4,546	12.4	≥30	M/F	MUFA	Age, sex, energy intake, serum lipids, systolic blood pressure, cigarette smoking, BMI, glucose intolerance	1 unit increase	9
Gardener et al. 2011 [43]	Northern Manhattan Study USA	Ischemic stroke Myocardial infarction Vascular death	2,568	9	>40	M/F	MUFA:SFA	Age, sex, race ethnicity, completion of high school education, moderate-to-heavy PA, energy intake, and cigarette smoking	≥ median vs. < median	7
Gillman et al. 1997 [40]	Framingham Heart Study USA	Stroke	832	20	45-65	M	MUFA	Age, energy, systolic blood pressure, cigarette smoking, glucose intolerance, BMI, PA, left ventricular hypertrophy, alcohol, fruit and vegetables	1% increase MUFA	9
Goldbourt et al. 1993 [18]	Israeli Ischemic Heart Disease Study ISR	CHD All-cause mortality	10,059	23	>40	M	MUFA:SFA	Age	Fifth vs. first quintile	7
Guasch-Ferre et al. 2014 [54]	PREvención con DIeta MEDiterránea SPA	All-cause mortality CVD mortality CVD events	7216	4.8	55-80	M/W	Olive oil	Age, sex, BMI, smoking status, alcohol intake, education level, PA, prevalence of diabetes, prevalence of hypertension, prevalence of hypercholesterolemia, use of antihypertensive, use of statins, Mediterranean diet adherence	Third vs. first tertile	8
He et al. 2003 [47]	Health professional study USA	Stroke	43,732	14	40-75	M	MUFA	BMI, PA, history of hypertension, smoking status, aspirin use, multivitamin use, and consumption of alcohol, potassium, fibre, and vitamin E, total servings of fruit and vegetables, total energy intake, and hypercholesterolemia at baseline	Fifth vs. first quintile	7
Houston et al. 2011 [38]	Health ABC study USA	CVD events	1,941	9	70-79	M/F	MUFA	Age, gender, race, education, field centre, smoking, alcohol use, PA, BMI, total energy intake, protein intake, fibre intake, multivitamin use, supplemental vitamin E use, statin use, aspirin use, oral estrogen use, and prevalent diabetes or hypertension, fat, PUFA, trans fat, and cholesterol	Third vs. first tertile	8
Iso et al. 2001 [37]	Nurses’ Health Study USA	Stroke	85,764	14	30-59	F	MUFA	Age, smoking status, time interval, BMI, alcohol intake, menopausal status and postmenopausal hormone use, vigorous exercise, usual aspirin use, multivitamin use, vitamin E use, n-3 fatty acid intake, calcium intake, and histories of hypertension, diabetes, high cholesterol levels, and total energy intake	Fifth vs. first quintile	7
Iso et al. 2003 [36]	JAP	Stroke	4,775	14	40-69	M/F	MUFA	Age, sex, quartiles of total energy intake and BMI, hypertension category, diabetes, serum total cholesterol, smoking status, ethanol intake, and menopausal status	Fourth vs. first quartile	8
Jakobsen et al. 2004 [4]	Multinational MONItoring of trends and determinants in CArdiovascular disease I, II EU	CHD	3,686	16	30-71	M/F	MUFA	Total energy intake, energy from protein, energy from fat, energy from carbohydrates, non-dietary and dietary coronary heart disease risk factors	5% increase	8
Kouris-Blazos et al. 1999 [55]	AUS	All-cause mortality	330	4-6	>70	M/F	MUFA:SFA	Age, sex and smoking status but not ethnic origin	MUFA:SFA (1 unit)	6
Larsson et al. 2012 [48]	Swedish Mammography Cohort SWE	Stroke	34,670	10.4	49-83	F	MUFA	Age, smoking status and pack-years of smoking, education, BMI, PA, history of hypertension, history of diabetes, aspirin use, family history of myocardial infarction, intakes of alcohol, protein, and dietary fibre, cholesterol	Fifth vs. first quintile	8
Lasheras et al. 2000 [26]	SPA	All-cause mortality	161	9	65-95	M/F	MUFA:SFA	Age, sex, BMI, albumin concentration, PA, self-assessment of health, and dieting for chronic conditions	MUFA:SFA (1 unit)	6
Leosdottir et al. 2007 [28], Wallström et al. 2012 [29]	Malmö Diet and cancer Study SWE	CVD events Stroke CHD	28,098	13.5	44-73	M/F	MUFA MUFA:SFA	Age, smoking habits, alcohol consumption, socioeconomic status, marital status, PA, BMI, fibre intake, and blood pressure, total fat intake for the ratio between unsaturated and saturated fats	Fifth vs. first quintile Fourth vs. first quartile	9
Levitan et al. 2013 [57]	Women’s Health Initiative trial and observational study USA	Heart Failure	68,132 (WHI) 93,676 (WHI-OS)	4.6	50-79	F	MUFA:SFA	Age at heart failure hospitalization, total energy intake, race/ethnicity, education, income, married, current smoking, total exercise, physical function, use of off-study postmenopausal hormone therapy, Women’s Health Initiative (WHI) study arm, systolic blood pressure, diastolic blood pressure, use of diuretics, β-blockers, and angiotensin converting enzyme inhibitors or angiotensin receptor blockers, BMI, and history of high cholesterol, high blood pressure, diabetes mellitus, myocardial infarction, coronary revascularization, and atrial fibrillation	Fourth vs. first quartile	8
Martinez-Gonzalez et al. 2011 [45]	Seguimiento University of Navarra SPA	All-cause mortality	15,535	6.8	University graduates (mean: 38)	M/F	MUFA:SFA	Age, years of university of education, BMI, smoking, PA, hours per day spent watching television, history of depression, baseline hypertension, baseline hypercholesterolemia, total energy intake, egg consumption, potato consumption, and adoption of special diets	≥ median vs. < median	8
Martinez-Gonzalez et al. 2009	Seguimiento University of Navarra SPA	CVD CHD	13,609	4.9	University graduates (mean: 38)	M/F	MUFA:SFA	Age, sex, family history of coronary heart disease, total energy intake, PA, smoking, BMI, diabetes at baseline, use of aspirin, history of hypertension and history of hypercholesterolemia	≥ median (W: ≥1.24, M: ≥1.19) vs. < median	8
Misirli et al. 2012 [52]	European Prospective Into Cancer and Nutrition GRE	Stroke Incidence Stroke Mortality	23,601	10.6	20-87	M/F	MUFA Olive Oil	Age, sex, education, smoking status, BMI, PA, hypertension, diabetes, and total energy intake.	Olive oil (23 g/d) MUFA (18 g/d)	9
Nagata et al. 2012 [31]	Takayama study JAP	All-cause mortality CVD mortality Cancer mortality	28,356	16	≥35	M/F	MUFA	Age, non-alcohol energy, and protein expressed as percentage of non-alcohol energy and was additionally adjusted for fat subtypes expressed as percentage of non-alcohol energy as appropriate, height, BMI, PA, smoking status, alcohol intake, education, marital status, menopausal status, histories of diabetes and hypertension, and intakes of fruits, vegetables, and dietary fibre	Fifth vs. first quintile	8
Oh et al. 2005 [33]	Nurses’ Health Study USA	CHD	78,778	20	30-55	F	MUFA	Age, BMI, smoking, alcohol intake, parental history of myocardial infarction, history of hypertension, menopausal status and hormone use, aspirin use, multivitamin use, vitamin E supplement use, PA, energy, protein, cholesterol intake, saturated, polyunsaturated, and trans-fat; a-linolenic acid; marine n-3 fatty acids; cereal fiber; and fruits and vegetables	Fifth vs. first quintile	7
Pietinen et al. 1997 [53]	Alpha-Tocopherol, Beta-Carotene Cancer Prevention Study USA	CVD events CVD mortality	21,930	6.1	50-69	M	MUFA Oleic acid	Age, smoking, BMI, blood pressure, energy intake, alcohol, education, PA	Fifth vs. first quintile	7
Posner et al. 1991 [46]	Framingham Study USA	CHD	813	16	45-65	M	MUFA	Energy, serum cholesterol levels, PA, systolic blood pressure, left ventricular hypertrophy, cigarette smoking, glucose intolerance, and metropolitan relative weight	For recommended vs. actual intake	9
Sauvaget et al. 2004 [35]	JAP	Stroke	3,731	14	35-89	M/F	MUFA	Age, sex, adjusted for radiation dose, city, BMI, smoking status, alcohol habits, and medical history of hypertension and diabetes	Third vs. first tertile	8
Samieri et al. 2011 [10]	Three city study FRA	Stroke	7,625	5.25	≥65	M/F	Olive oil	Age, sex, education, smoking status, BMI, PA, hypertension, diabetes, and total energy intake	Third vs. first tertile	8
Solfrizzi et al. 2005 [51]	Italian Longitudinal Study on Aging ITA	All-cause mortality	278	8.5	65-84	F	MUFA MUFA:SFA	Age, sex, waist-hip ratio, smoking status, Charlson co-morbidity index, and total energy intake	Fourth vs. first quartile	7
Tognon et al. 2011 [24]	DEN	All-cause mortality	1,037	8.5	70	M/F	MUFA:SFA	Gender, BMI, waist circumference, PA, smoking status, marital status and education	≥ median vs. < median	7
Trichopoulou et al. 2005 [23]	European Prospective Into Cancer and Nutrition Elderly EU	All-cause mortality	74,607	7.4	>60	M/F	MUFA MUFA:SFA	Age, sex, diabetes mellitus at baseline, waist to hip ratio, BMI, educational achievement, smoking status, PA at occupation, PA score at leisure, alcohol intake, and total energy intake	MUFA (12 g) MUFA:SFA (0.4)	8
Trichopoulou et al. 2009 [20]	European Prospective Into Cancer and Nutrition GRE	All-cause mortality	23,349	8.5	20-86	M/F	MUFA:SFA	Age, sex, education, smoking status, waist-to-hip ratio, BMI, MET score, and total energy intake	≥ median vs. < median	9
Trichopoulou et al. 1995 [22]	GRE	All-cause mortality	182	4-5	>70	M/F	MUFA:SFA	Age, sex, and smoking status	MUFA:SFA (1 unit)	7
Trichopoulou et al. 2003 [21]	European Prospective Into Cancer and Nutrition GRE	All-cause mortality	22,043	3.7	20-86	M/F	MUFA Olive oil MUFA:SFA	Age, sex, waist-to-hip ratio, energy-expenditure score, years of education, smoking status, BMI, and total energy intake	MUFA (15 g/d) Increment olive oil (20 g) MUFA:SFA (0.5)	9
Van den Brandt et al. 2011 [27]	Netherlands cohort study NED	All-cause mortality	120,852	10	55-69	M/F	MUFA:SFA	Age, cigarette smoking status, number of cigarettes smoked per day, years of smoking, BMI, non-occupational PA, history of hypertension, highest level of education, and energy intake	Fourth vs. first quartile	9
Yaemsiri et al. 2012 [39]	Women’s Health initiative observational study USA	Stroke	87,025	7.6	50-79	F	MUFA	Age, race, education, family income, total metabolic equivalent task hours per week, alcohol intake, history of CHD, history of atrial fibrillation, history of diabetes, aspirin use, use of antihypertensive medication, use of cholesterol-lowering medication, BMI, systolic blood pressure, and total energy intake, dietary vitamin E, fruits and vegetable intake, fibre	Fifth vs. first quintile	8
Xu et al. 2006 [41]	Strong Heart Study USA	CHD CHD mortality	2,938	7.2	47-79	M/F	MUFA	Age, sex, energy, study centre, diabetes status, BMI, HDL, LDL, triacylglycerol, smoking, alcohol consumption, hypertension, percentage of energy from protein, and total energy intake	Fourth vs. first quartile	9
Wakai et al. 2014 [30]	Japan Collaborative Cohort Study JAP	All-cause mortality CVD mortality	58,672	19.3	40-79	M/F	MUFA	Age, area, education, smoking, alcohol consumption, BMI, sleep duration, walking, consumption of vegetables and fruit, and total energy intake	Fifth vs. first quintile	8

*BMI* Body Mass Index, *CHD* coronary heart disease, *CVD* cardiovascular disease, *DEN* Denmark, *EDI* Elderly Diet Index, *EU* European Union, *FRA* France, *GBR* Great Britain, *HDI* Healthy Diet Index, *ISR* Israel, *ITA* Italy, *JAP* Japan, *MET* metabolic equivalent of task, *MUFA* monounsaturated fatty acids, *NED* The Netherlands, *PA* physical activity, *SFA* saturated fatty acids, *SPA* Spain, *SWE* Sweden, *WHI* Women’s Health Initiative, *USA* United States of America.

### Main outcomes

According to the different clinical outcomes, overall risk of all-cause mortality was evaluated in seventeen cohorts, cardiovascular mortality in fourteen cohorts, combined cardiovascular events in twenty-eight cohort studies, coronary heart disease in fifteen cohorts, and stroke in eleven cohorts, respectively.

**Figure 1:**
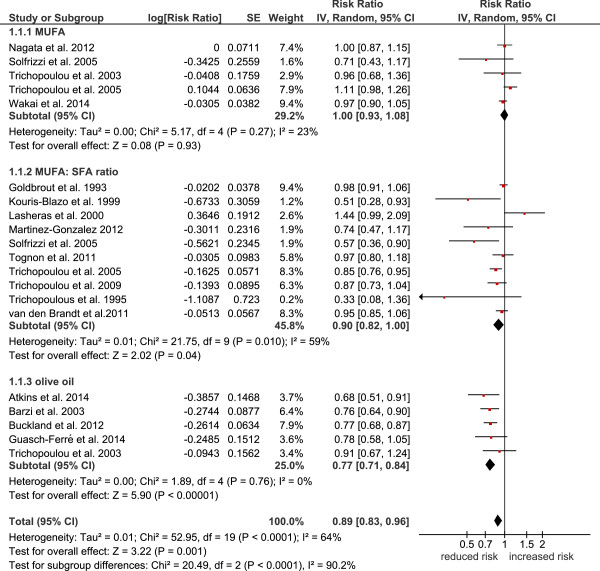
**Forest plot showing pooled relative risks (RRs) with 95% CI for all-cause mortality comparing the top versus bottom third of the distribution of MUFA, MUFA:SFA ratio, olive oil, and oleic acid.** I^2^: Inconsistency; MUFA: monounsaturated fatty acids; SE: standard error; SFA: saturated fatty acids.

**Figure 2:**
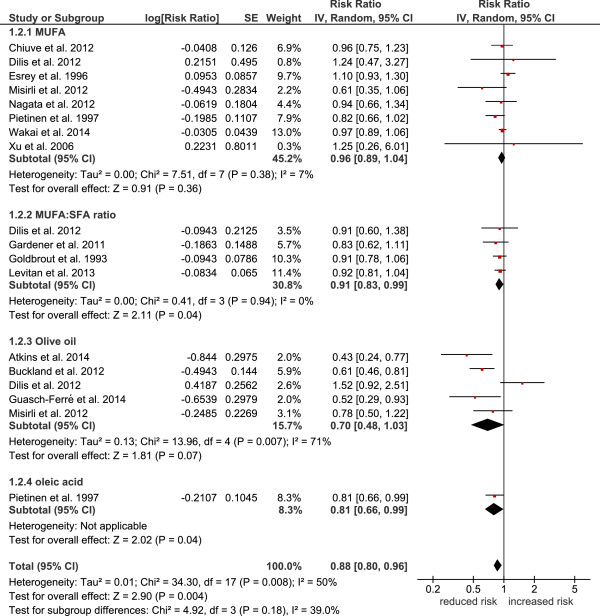
**Forest plot showing pooled relative risks (RRs) with 95% CI for cardiovascular mortality comparing the top versus bottom third of the distribution of MUFA, MUFA:SFA ratio, olive oil, and oleic acid.** I^2^: Inconsistency; MUFA: monounsaturated fatty acids; SE: standard error; SFA: saturated fatty acids.

**Figure 3:**
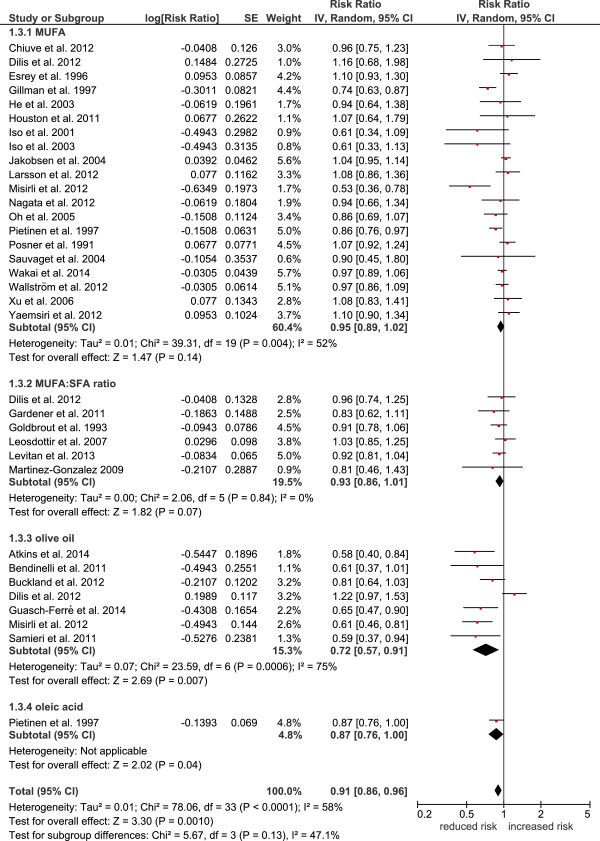
**Forest plot showing pooled relative risks (RRs) with 95% CI for combined cardiovascular events comparing the top versus bottom third of the distribution of MUFA, MUFA:SFA ratio, olive oil, and oleic acid.** I^2^: Inconsistency; MUFA: monounsaturated fatty acids; SE: standard error; SFA: saturated fatty acids.

Random effects model data (as summarized in Table 2) revealed that top versus bottom third combined MUFA, olive oil, oleic acid, and MUFA:SFA ratio was significantly associated with a reduced risk of: all-cause mortality (relative risk, RR: 0.89, 95% confidence interval 0.83 to 0.96; p = 0.001, I^2^ = 64%) (Figure 1), cardiovascular mortality (RR: 0.88, 95% CI 0.80 to 0.96, p = 0.004, I^2^ = 50%) (Figure 2), combined cardiovascular events (RR: 0.91, 95% CI 0.86 to 0.96, p = 0.001, I^2^ = 58%) (Figure 3), and stroke (RR: 0.83, 95% CI 0.71 to 0.97, p = 0.02, I^2^ = 70%). In contrast, no significant changes could be observed for coronary heart disease (RR: 0.96, 95% CI 0.90 to 1.01, p = 0.13, I^2^ = 41%).

**Table 2:** **Relative risk for all-cause mortality, cardiovascular mortality, combined cardiovascular events, stroke, and coronary heart disease (with 95% confidence intervals) comparing the top versus bottom third of MUFA, MUFA:SFA ratio, olive oil, and oleic acid**

Outcome	No studies	MUFA source	Relative risk	95% CI	p-value	I ^2^ (%) ^a^ 95% CI
All-cause mortality	17	All MUFA combined	0.89	0.83 to 0.96	0.001	64
						42 to 78
	5	MUFA	1.00	0.93 to 1.08	0.93	23
	10	MUFA:SFA	0.90	0.82 to 1.00	0.04	59
	5	Olive oil	0.77	0.71 to 0.84	<0.00001	0
Cardiovascular mortality	14	All MUFA combined	0.88	0.80 to 0.96	0.004	50
						15 to 71
	8	MUFA	0.96	0.89 to 1.04	0.36	7
	4	MUFA:SFA	0.91	0.83 to 0.99	0.04	0
	5	Olive oil	0.70	0.48 to 1.03	0.07	71
	1	Oleic acid	0.81	0.66 to 0.99	0.04	n.a.
Combined cardiovascular events	30	All MUFA combined	0.91	0.86 to 0.96	0.001	58
						38 to 71
	20	MUFA	0.95	0.89 to 1.02	0.14	52
	6	MUFA:SFA	0.93	0.86 to 1.01	0.07	0
	7	Olive oil	0.72	0.57 to 0.91	0.007	75
	1	Oleic acid	0.87	0.76 to 1.00	0.04	n.a.
Stroke	11	All MUFA combined	0.83	0.71 to 0.97	0.02	70
						46 to 84
	9	MUFA	0.85	0.72 to 1.01	0.07	65
	1	MUFA:SFA	1.18	0.91 to 1.53	0.21	n.a.
	2	Olive oil	0.60	0.47 to 0.77	<0.0001	0
Coronary heart disease	15	All MUFA combined	0.96	0.90 to 1.01	0.13	41
						0 to 66
	9	MUFA	0.99	0.93 to 1.06	0.76	29
	4	MUFA:SFA	0.94	0.86 to 1.02	0.14	0
	4	Olive oil	0.80	0.57 to 1.14	0.22	77
	1	Oleic acid	0.87	0.76 to 1.00	0.04	n.a.

^a^ *I* ^*2*^ inconsistency, percentage of variation across studies due to heterogeneity.

*MUFA* monounsaturated fatty acids, *n.a.* not applicable, *SFA* saturated fatty acids.

### Subgroup/sensitivity analyses

Following subgroup analyses, olive oil most likely turned out to be crucial for the results of the primary analysis, since significant associations could only be found between higher intakes of olive oil and reduced risk of all-cause mortality (RR: 0.77, 95% CI 0.71 to 0.84, p < 0.00001, I^2^ = 0%), cardiovascular events (RR: 0.72, 95% CI 0.57 to 0.91, p = 0.007, I^2^ = 77%), and stroke (RR: 0.60, 95% CI 0.47 to 0.77, p < 0.0001, I^2^ = 0%), respectively. Subgroup analysis for MUFA (of mixed animal and plant origin) did not reveal any significant risk reduction for all-cause mortality (RR: 1.00, 95% CI 0.93 to 1.08, p = 0.93, I^2^ = 23%), cardiovascular mortality (RR: 0.95, 95% CI 0.89 to 1.02, p = 0.14, I^2^ = 52%), cardiovascular events (RR: 0.96, 95% CI 0.89 to 1.04, p = 0.36, I^2^ = 7%), coronary heart disease (RR: 0.99, 95% CI 0.93 to 1.06, p = 0.76, I^2^ = 29%), and stroke (RR: 0.85, 95% CI 0.72 to 1.01, p = 0.07, I^2^ = 65%). To investigate statistical heterogeneity, sensitivity analyses were performed with the *metaan* command in STATA. Heterogeneity of the main analysis could be confirmed in the sensitivity analyses. Differentiating between studies performed in Europe vs. non-European investigations resulted in significant differences as compared to the main analysis. Pooling European based cohorts resulted in a significant risk reduction for all-cause mortality (RR: 0.87, 95% CI 0.79 to 0.95) as well as for cardiovascular mortality (RR: 0.76, 95% CI 0.64 to 0.91) and cardiovascular events (RR: 0.86, 95% CI 0.78, 0.95). In contrast, no significant reduction in all-cause mortality risk (RR: 0.97, 95% CI 0.91 to 1.04) could be observed for non-European cohorts (the respective data for cardiovascular mortality being RR: 0.94, 95% CI 0.89 to 0.99 and for cardiovascular events being RR: 0.93, 95% CI 0.87 to 0.98). With respect to study length, studies with a follow-up ≥ 10 years resulted in similar results as compared to short-term studies (<10 years follow up). Likewise, high quality studies could confirm the results of the primary analysis.

### Publication bias

The Egger’s linear regression tests provided evidence for a potential publication bias for combined cardiovascular events (p = 0.018), all-cause mortality (p = 0.041), and cardiovascular mortality (p = 0.12) following comparison of the top versus bottom third combined MUFA, olive oil, oleic acid, and MUFA:SFA ratio. No evidence of publication bias could be detected for risk of CHD (p = 0.28) and stroke (p = 0.28). All funnel plots indicate little to moderate asymmetry, suggesting that publication bias cannot be completely excluded as a factor of influence on the present meta-analysis (Additional file 1: Figures S1, S2, S3, S4 and S5). It remains possible that small studies with inconclusive results have not been published or failed to do so.

## Discussion

In the present meta-analysis, comparison of the top versus the bottom third of combined MUFA subgroups (MUFA, olive oil, oleic acid, and MUFA:SFA) was associated with reduced risk of all-cause mortality (11%), cardiovascular mortality (12%), combined cardiovascular events (9%), and stroke (17%). In the ensuing subgroup analyses, this significant correlation could only be observed between higher intakes of olive oil and reduced risk of all-cause mortality, cardiovascular events, and stroke, respectively. In contrast, monounsaturated fatty acids of mixed animal and plant origin did not result in any significant effects with respect to these outcome parameters. Thus, it seems possible that olive oil represents the crucial factor of influence for the protective health effects observed in the primary analysis. However, one has to keep in mind the limitations of the present systematic review and meta-analysis summarized at the end of this section, especially the fact that the specific sources of MUFA have not been indicated in every study.

In order to properly evaluate the potential beneficial or detrimental effects of MUFA with respect to cardiovascular diseases, it seems of importance to consider the source of food providing these fatty acids. In the Nurses’ Health Study, MUFA intake was highly correlated with SFA intake (correlation coefficient of 0.81) but only moderately correlated with intakes of PUFA (correlation coefficient of 0.30), suggesting that fat was primarily of animal origin [58]. In the different EPIC cohorts, MUFA intakes ranged between approximately 10% of daily total energy consumption (TEC) in The Netherlands and ~ 20% of TEC in Greece. In general, intake of MUFA was higher in southern European countries as compared central or northern cohorts. However, another distinguishing feature seems to be the predominant source of MUFA in the respective cohorts. In Greece, Spain, and Italy, fat of plant origin (mainly olive oil) provided up to 64% of MUFA intake, whereas in most other EPIC centers, the main contributors to total MUFA intake were meat and meat products, added fats, and dairy products [8]. This might also provide an explanation for the somewhat mixed results provided by systematic reviews and meta-analyses in the past. Thus, a diet rich in MUFA was found to have beneficial effects on a broad range of CVD risk factors, not only in the primary prevention of CVD [1, 59–61]. On the other hand, no association between total and individual MUFA and CHD was reported in a meta-analysis of studies assessing both dietary intake and circulating fatty acid composition, [7] while a meta-analysis of observational studies suggested that replacing SFAs with PUFAs might have a greater benefit than replacement of SFAs by MUFA [4]. There is some evidence drawn from prospective studies of an adverse association between MUFA and coronary events, but this correlation might be influenced by high amounts of MUFA of animal origin [4].

A number of *in-vivo* and *in-vitro* studies examined the health effects of extra virgin olive oil, the potential “Unique Selling Proposition” of a genuine Mediterranean diet. Thus, the Di@bet.es study demonstrated that individuals who consumed olive oil had a significantly lower risk of developing obesity, impaired glucose metabolism, hypertriglyceridemia, and lower HDL cholesterol levels as compared to a group consuming sunflower oil [62]. In addition, results from experimental studies provide evidence that olive oil consumption improves several CHD risk factors [63, 64]. The PREDIMED dietary intervention trial aimed a intake of 50 g/d or more of extra virgin olive oil observed a significant risk reduction of both combined cardiovascular events as well as primary stroke, but not of CHD, indicating a consistency with the results of the present meta-analyses of cohort studies [9]. In a long-term intervention trial by Esposito et al., a higher regression in as well as a lower rate of progression of the intima–media thickness of the carotid artery was found in the group adopting a Mediterranean diet as compared to a low-fat diet reference arm [65].

Apart from oleic acid, olive oil contains a number of bioactive compounds such as polyphenols which are especially prominent in virgin and extra-virgin olive oil, but not in refined olive oil [64, 66]. A key olive oil polyphenol is oleuropein (a compound that generates tyrosol and hydroxytyrosol), which accounts for approximately 80% of olive oil phenolic content and is a potent scavenger of superoxide radicals and inhibits LDL oxidation [67, 68]. There is a causal link between oxidative stress, inflammation, endothelial dysfunction, and CVD/CHD [69]. A meta-analysis of intervention trials provide evidence that an MD decreases inflammation and improves endothelial function [70]. When focusing on virgin olive oil consumption, the inverse correlation between olive oil and CHD risk found in the present meta-analysis is consistent with the fact that olive oil is not just a supplier of MUFA but of other biologically active components as well.

Several limitations should be taken into account when interpreting the results of the present meta-analysis. MUFA coexist with SFA in several food sources. In addition, cis- and trans-isomers of MUFAs were sometimes categorized together in cohort studies. Furthermore, moderate to substantial heterogeneity could be observed in the present meta-analysis. Potential sources of heterogeneity include combining MUFA/olive oil/oleic acid/MUFA:SFA ratio in the same analysis, heterogeneous risk estimates, heterogeneous populations/ages/gender, sample sizes as well as follow-up periods of the included studies. No unpublished data were considered for the present meta-analysis, and it cannot be excluded that these results may influence the effect size estimates. Examination of funnel plots showed little to moderate asymmetry suggesting that publication bias cannot be completely excluded as a confounder of the present meta-analysis (e.g. it remains possible that small studies yielding inconclusive data have not been published). In addition, the specific food sources of MUFA could not always identified, limiting the validity of any general recommendation towards MUFAs of plant origin (it is most likely olive oil, but it might be other types of food as well, e.g. nuts, canola oil or a specific variety of sunflower oil). Conversely, it might be that results from studies using mixed sources of MUFA might be biased by non-identified olive oil, making MUFA appear to be beneficial in general when some sources are not. Furthermore, observational studies including cohort studies assessing outcome events affected by nutrition should be interpreted with caution, since reliance on nutritional assessment methods with validity and reliability is lower when compared to randomized controlled trials.

However, the present study has some complementary strengths as well. Compared to cohort studies, dietary intervention trials are often limited by lack of double blinding, non-compliance, cross-over, and high drop-out rates. Therefore, well-designed analyses in prospective cohort studies could also provide important evidence with respect to long-term clinical outcomes. Another strength of this work is the inclusion of an overall population >800,000 subjects. To the best of our knowledge, this represents the most comprehensive summary of the evidence on MUFA, olive oil, MUFA:SFA on hard clinical outcome parameters.

## Conclusion

The results of the present meta-analysis indicate an overall risk reduction of all-cause mortality (11%), cardiovascular mortality (12%), cardiovascular events (9%), and stroke (17%) when comparing the top vs. bottom thirds of a combination of MUFA, olive oil, oleic acid, and MUFA:SFA ratio. Monounsaturated fat of mixed animal and vegetable sources per se did not yield any significant effects on these outcome parameters. Subgroup analysis indicated that only olive oil (the primary monounsaturated fat source in south European countries) is was associated with a significant risk reduction for several outcomes. These data provide evidence that the source and origin of MUFA within a specific diet should be taken into account in order to evaluate the potential benefits of this type of fatty acids. Further studies are required evaluating specific food sources of MUFA and risk of all-cause mortality and CVD events.

## Electronic supplementary material

Additional file 1: **Detailed search strategy; Figure S1-S5: Funnel Plots.** (DOCX 44 KB)
