# Supplementary material for: Monounsaturated fatty acids, olive oil and health status: a systematic review and meta-analysis of cohort studies
Source: Lipids Health Dis. 2014 Oct 1;13:154. doi: 10.1186/1476-511X-13-154 (PMC4198773; doi:10.1186/1476-511X-13-154)
Supplement: Supplementary file 1 — Additional file 1: Detailed search strategy; Figure S1-S5: Funnel Plots. (DOCX 44 KB) [file 12944_2014_1138_MOESM1_ESM.docx]

**Additional file 1**

**Detailed search strategy: PUBMED (02.06.2013); 2000 results**

("dietary fat"[All Fields] OR "fatty acids"[All Fields] OR "monounsaturated fat"[All Fields] OR "mufa"[All Fields] OR "olive oil"[All Fields] OR "oleic acid"[All Fields] OR "mediterranean diet"[All Fields]) AND ("cardiovascular disease"[All Fields] OR "myocardial infarction"[All Fields] OR "coronary heart disease"[All Fields] OR "stroke"[All Fields] OR "mortality"[All Fields]) AND ("incidence"[All Fields] OR "cohort"[All Fields] OR "follow-up"[All Fields] OR "prospective"[All Fields] OR "risk ratio"[All Fields] OR "hazard ratio"[All Fields] OR "rate ratio"[All Fields])


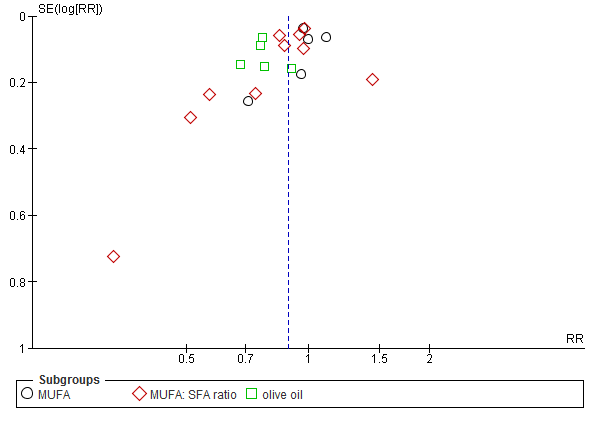


Figure S1. Funnel plot showing study precision against the relative risk with 95% CIs for all-cause mortality. SE = Standard error


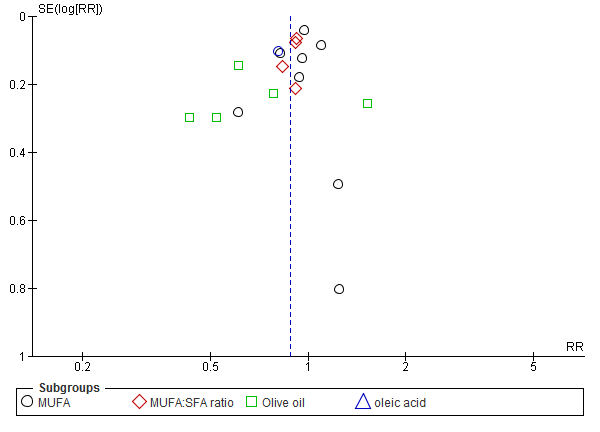


Figure S2. Funnel plot showing study precision against the relative risk with 95% CIs for cardiovascular mortality. SE = Standard error


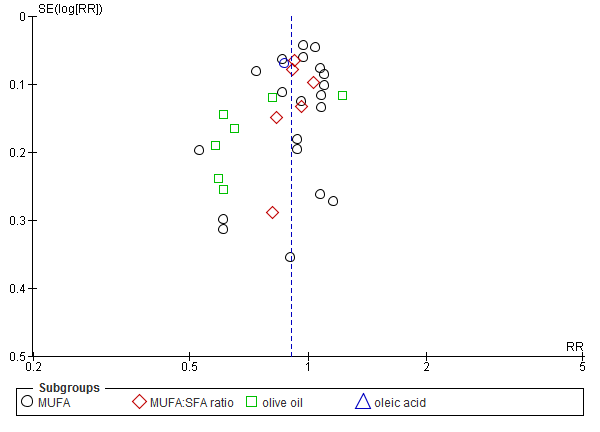


Figure S3. Funnel plot showing study precision against the relative risk with 95% CIs for combined cardiovascular events. SE = Standard error


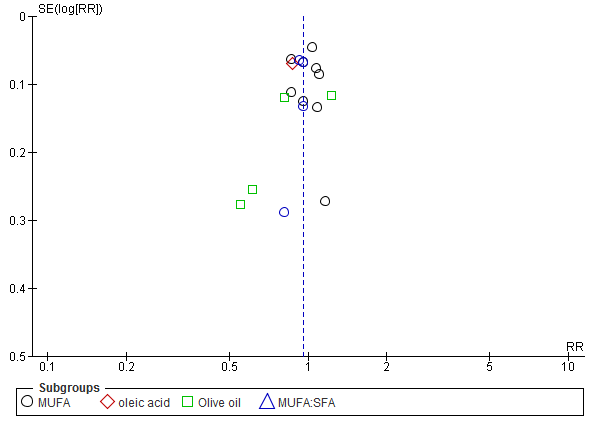


Figure S4. Funnel plot showing study precision against the relative risk with 95% CIs for coronary heart disease. SE = Standard error


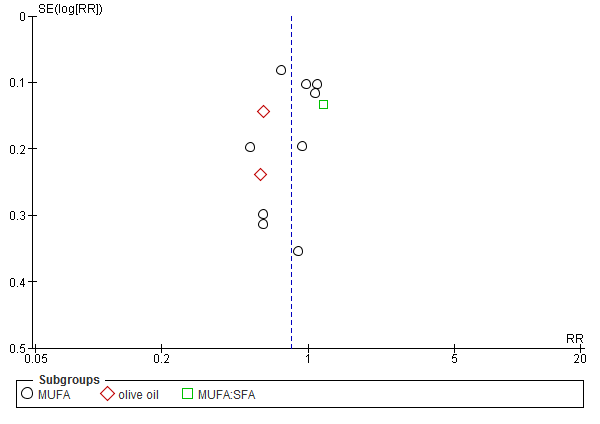


Figure S5. Funnel plot showing study precision against the relative risk with 95% CIs for stroke. SE = Standard error
